# Photoperiodic Stress in Eggplant and Tomato: Growth Retardation and Leaf Disorders

**DOI:** 10.3390/plants15172695

**Published:** 2026-09-02

**Authors:** Tatjana G. Shibaeva, Ilya A. Levkin, Elena G. Sherudilo, Alexandra A. Rubaeva, Irina A. Nilova, Alexander F. Titov

**Affiliations:** Institute of Biology, Karelian Research Center, Russian Academy of Sciences, Petrozavodsk 185910, Russia; levkin-556@mail.ru (I.A.L.); sherudil@krc.karelia.ru (E.G.S.); arubaeva@krc.karelia.ru (A.A.R.); im-ira@mail.ru (I.A.N.); titov@krc.karelia.ru (A.F.T.)

**Keywords:** eggplant, light-dark cycle, photoperiodic stress, tomato

## Abstract

In controlled-environment agriculture systems, such as vertical farms, plants can be cultivated under artificial light-dark (L/D) cycles that deviate substantially from the natural 24 h photoperiod. While these regimes may enhance the growth and quality of certain crops, the physiological response is highly species-specific, and long-term implications for many crop species remain insufficiently characterized. This study aimed to evaluate the effects of a shortened L/D cycle (8/4 h, repeated twice daily) on the morphology, photosynthetic activity, and redox status of eggplant (*Solanum melongena* L., cv. ‘Almaz’) and tomato (*Solanum lycopersicum* L., cv. ‘Verlioka Plus’) seedlings, compared to a standard 16/8 h photoperiod. Plants were grown in chambers equipped with light-emitting diode (LED) and fluorescent (FLU) lamps under an equivalent daily light integral (DLI: 11.5 mol m^−2^ day^−1^). The shortened 8/4 h L/D cycle induced significant growth suppression, reduced leaf area, and decreased the maximum quantum yield of photosystem II (*Fv*/*Fm*), as well as chlorophyll and carotenoid content in both species. Under this regime, eggplants exhibited leaf chlorosis accompanied by necrotic lesions, whereas tomato plants displayed interveinal chlorosis. Notably, significant accumulation of oxidative stress markers—a 37% increase in malondialdehyde (MDA) and a 30% elevation in H_2_O_2_ levels—occurred exclusively in eggplant. These findings demonstrate that redistributing the same 16 h daily illumination into two 8/4 h L/D cycles induces stress in both eggplant and tomato plants, with eggplant exhibiting greater injury than tomato. The underlying circadian mechanisms and the apparent differences between LED and FLU lighting require further controlled validation.

## 1. Introduction

In plants, the light/dark (L/D) cycle is the most important cyclic environmental factor. These cycles are characterized by two key parameters: the total cycle length, or period, and the ratio of light to dark periods (the L/D ratio). A 24 h L/D cycle, matching the natural diurnal period, is considered normal. Cycles that deviate from this 24 h period are classified as abnormal [1,2]. The use of abnormal (non-standard) L/D cycles is permissible in controlled-environment agriculture systems, such as plant factories or vertical farms. In these environments, external environmental cues are absent, and the natural 24 h diurnal cycle is not imposed [3]. Recent studies have shown that non-standard L/D cycles—whether shortened or extended—generated by artificial lighting can enhance plant growth and development. For example, lettuce (*Lactuca sativa*) and basil (*Ocimum basilicum*) demonstrated significantly higher yields under shortened L/D cycles compared with standard 24 h photoperiods [4,5]. Exposure of spring wheat (*Triticum aestivum*) to a 6/6 h L/D cycle significantly enhanced plant growth and development relative to the standard 12/12 h photoperiod [6,7]. Both theoretical suggestions and experimental evidence indicate that abnormal L/D cycles can enhance the production of valuable secondary metabolites in plants [8,9]. For example, in lettuce, an 8/4 h lighting regime significantly increased levels of soluble protein, soluble sugars, and carotenoids relative to a standard 16/8 h photoperiod. In contrast, no such changes were observed under a 12/6 h regime [10]. Consequently, selecting an appropriate L/D cycle for specific crops may offer economic and environmental benefits in controlled environment agriculture (CEA) [9,10,11]. Multiple L/D cycles offer a promising strategy to reduce costs in vertical farming systems, particularly when synchronized with real-time electricity pricing. By applying maximum light intensity during low-cost periods and minimizing or eliminating lighting during peak hours, significant economic benefits can be achieved. However, this optimization approach requires careful evaluation of potential trade-offs, as multiple L/D cycles within a 24 h period may reduce plant growth—a factor that must be balanced against energy savings [12].

The ability of plants to respond appropriately to L/D cycles is a crucial evolutionary adaptation. The circadian clock acts as an internal timing mechanism, synchronizing numerous physiological and biochemical processes and enabling plants to anticipate and adapt to environmental fluctuations such as the daily L/D cycle. However, frequent alterations in external L/D cycles can disrupt circadian rhythmic gene expression, potentially compromising plant performance and overall fitness [10]. In *Arabidopsis*, circadian genes adjust their oscillation patterns to align with the duration of the external L/D cycle. This synchronization enhances photosynthesis and improves plant survival [13]. Similarly, in tomato (*Solanum lycopersicum*), circadian genes *EID1* and *LNK2* respond positively to various L/D cycles. This suggests a conserved adaptive mechanism across different plant species [14]. Most studies examining the effects of abnormal L/D cycles have focused on lettuce. In one such study, researchers conducted a comparative analysis of gene expression levels under 16/8 h, 12/6 h, and 8/4 h L/D cycles, revealing 7209 differentially expressed genes [10]. Furthermore, weighted gene co-expression network analysis (WGCNA) identified three gene modules closely associated with the L/D cycle. This suggests coordinated regulation of specific biological pathways in response to photoperiod variations. The eigengenes of these modules were significantly enriched in key pathways, including plant hormone signal transduction, sphingolipid metabolism, and nucleocytoplasmic transport. Network analysis further identified six hub genes—*CIP1*, *SCL34*, *ROPGEF1*, *ACD6*, *CcmB*, and *Rps4*—within the modules. These genes play key roles in regulating plant circadian rhythms and significantly influence lettuce growth. Real-time quantitative PCR (qPCR) analysis confirmed that their diurnal expression patterns varied significantly under different L/D cycles. [10]. Thus, the accumulating body of data now enables a comprehensive understanding of the mechanisms underlying plant responses to abnormal L/D cycles.

Vertical farms are optimized for growing highly profitable, fast-growing, and compact crops. The key categories include leafy greens, microgreens, herbs, berries, and compact vegetable crops. Current trends in industrial agriculture are driving the development of hybrid cultivation systems, which synergistically combine indoor vertical farming with traditional greenhouse technologies. This “division of labour” involves vertically propagating tomato, pepper, cucumber, and other vegetable seedlings under controlled artificial lighting before transferring them to traditional greenhouses for the final growth stages and fruit production. This integrated approach optimizes resource use by combining the strengths of both systems: precise control during early growth stages in vertical farms and cost-effective, large-scale cultivation in greenhouses.

Based on the above, the primary objective of this study was to examine the effects of an 8/4 h L/D cycle under light-emitting diode (LED) and fluorescent (FLU) lighting systems on key physiological and biochemical parameters in eggplant (*Solanum melongena*) and tomato (*Solanum lycopersicum*) during the seedling stage.

## 2. Results

### 2.1. Plant Growth and Development

To evaluate the effects of abnormal L/D cycles on plant phenotype, we measured several morphological and biomass parameters, including plant height, stem diameter, and fresh (FW) and dry (DW) weight of leaves and stems. The results show that plant phenotype was significantly altered under different L/D cycles (Table 1 and Table 2).

**Figure 1:**
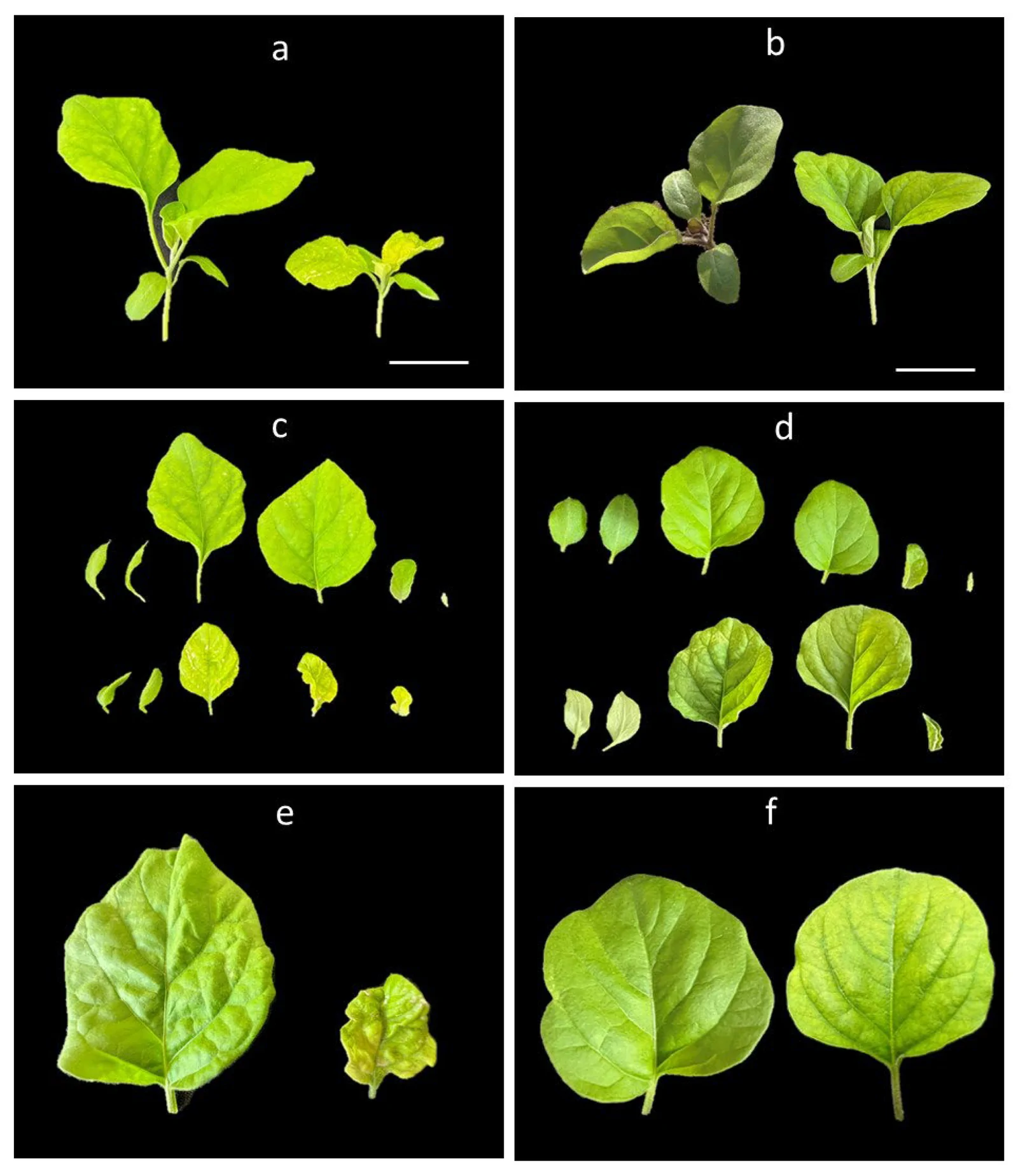
Eggplant (*Solanum melongena* L.) morphology on day 17. Panels: (**a**,**b**) whole plants; (**c**,**d**) all leaves; (**e**,**f**) second true leaves. Light/dark cycles: left/top, 16/8 h (control); right/bottom, 8/4 h. Light sources: LED (**a**,**c**,**e**), FLU (**b**,**d**,**f**) (scale bar = 5 cm).

**Figure 2:**
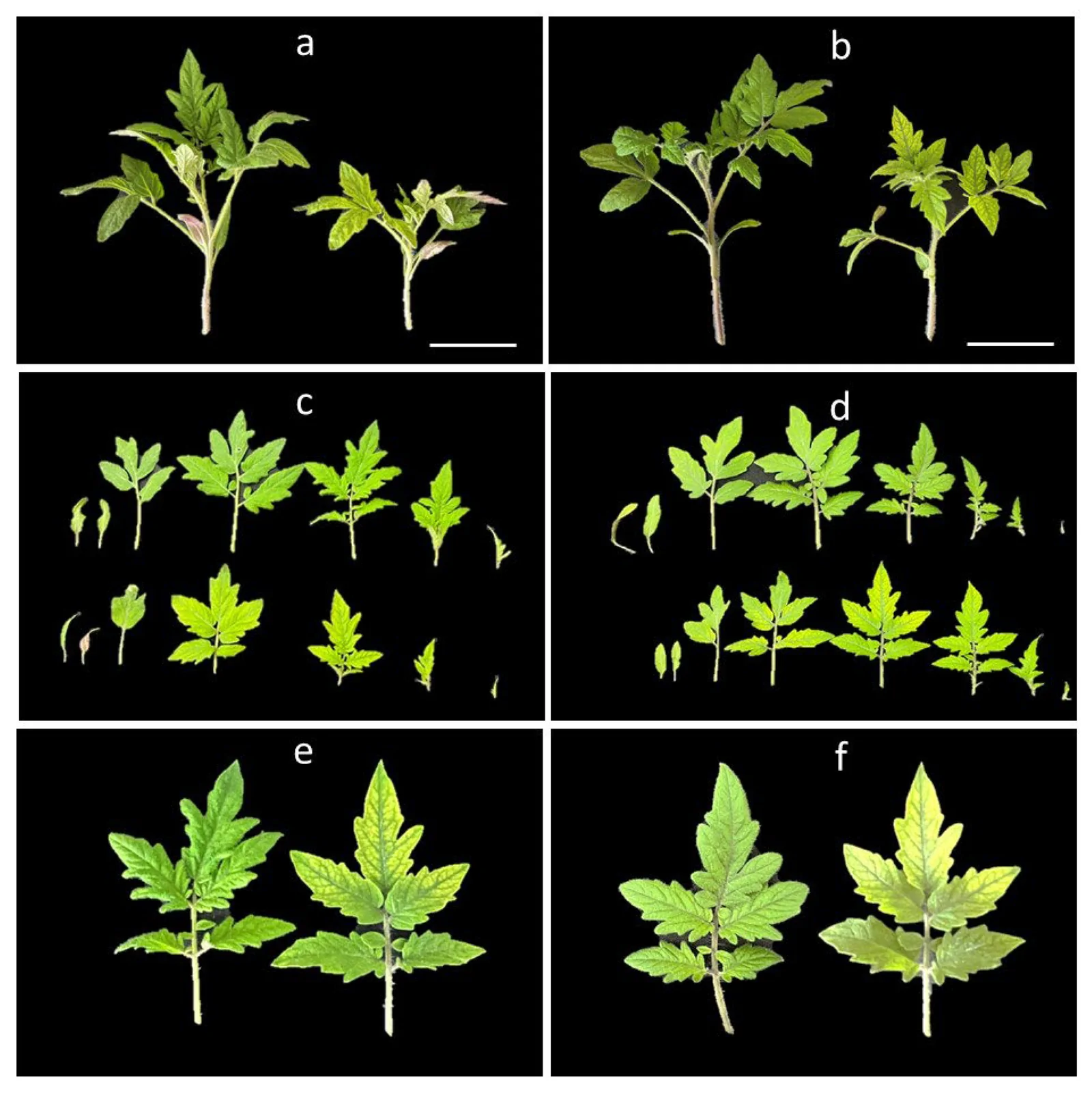
Tomato (*Solanum lycopersicum* L.) morphology on day 21. Panels: (**a**,**b**) whole plants; (**c**,**d**) all leaves; (**e**,**f**) second true leaves. Light/dark cycles: left/top, 16/8 h (control); right/bottom, 8/4 h. Light sources: LED (**a**,**c**,**e**), FLU (**b**,**d**,**f**) (scale bar = 5 cm).

**Figure 3:**
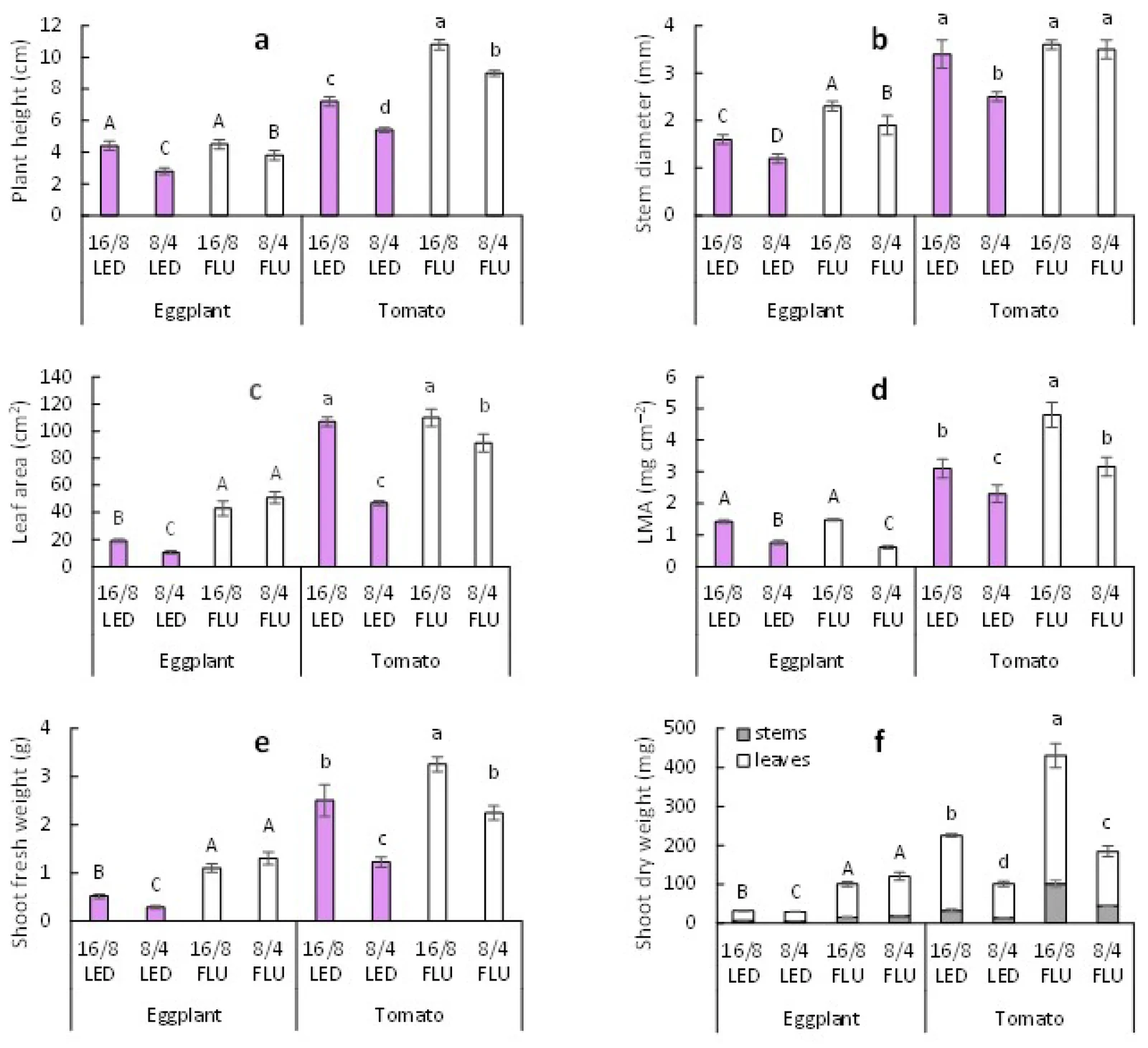
Influence of L/D cycles (16/8 h (control) vs. 8/4 h) and light source type (LED (purple bars) vs. FLU (white bars)) on morphological and biomass-related traits in eggplant (*Solanum melongena* L.) and tomato (*Solanum lycopersicum* L.): (**a**) plant height, (**b**) stem diameter, (**c**) leaf area, (**d**) leaf mass per area (LMA), (**e**) shoot fresh weight (FW), (**f**) shoot dry weight (DW). Statistically significant differences between treatments within each species are denoted by different letters (capital letters for eggplant, small letters for tomato) (*p* < 0.05). Values are expressed as mean ± standard error, *n* = 20.

Both eggplant and tomato plants grown under an 8/4 h L/D cycle with both FLU and LED lighting exhibited reduced plant height (16–36%) compared to those exposed to a standard 16/8 h photoperiod (Figure 1a,b, Figure 2a,b and Figure 3a). Similarly, stem diameter was smaller by 17–26% across all 8/4 h treatments, with the exception of tomato plants grown under FLU lamps (Figure 3b). Furthermore, the 8/4 h L/D cycle significantly reduced leaf area by 16–56%; shoot FW by 31–51%; shoot DW by 9–57%, in all treatments except eggplants grown under FLU lamps (Figure 1c,d, Figure 2c,d and Figure 3c,e,f).

The lighting regime had no significant effect on biomass partitioning between leaves and stems; however, the type of light source did influence this parameter in tomato plants.

Specifically, tomato plants grown under FLU lamps allocated a higher proportion of aboveground biomass to leaves (86%) compared to those grown under LED lighting (76–77%). Conversely, the stem proportion was greater under LEDs (23–24%) than under FLU lamps (14%) (Figure 3f).

Leaf mass per area (LMA) was reduced by 25–58% across all plants exposed to the 8/4 h L/D cycle, compared to those under the standard 16/8 h cycle (Figure 3d).

### 2.2. Leaf Disorders

**Figure 4:**
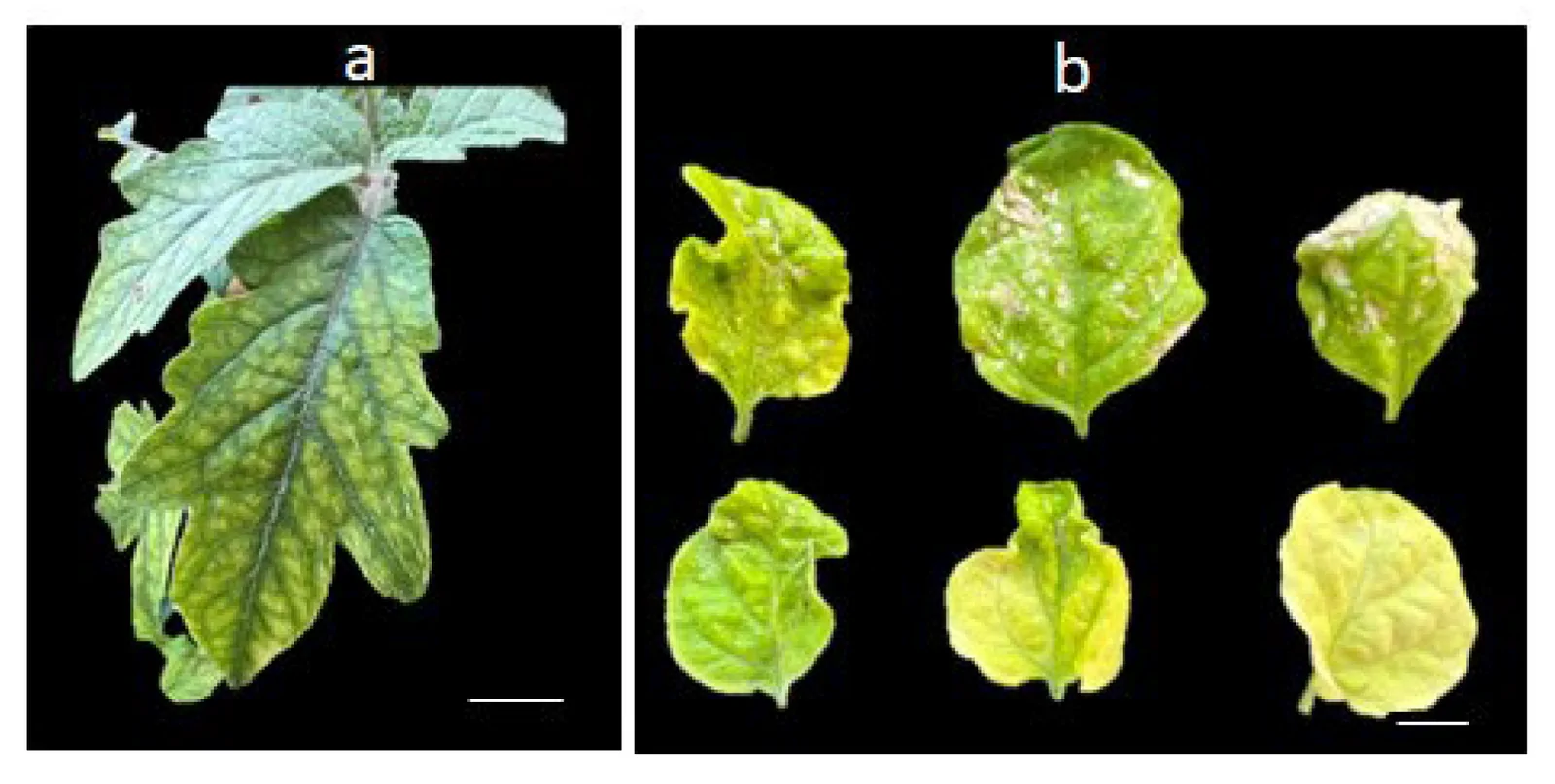
Leaf photoinjuries under the 8/4 h L/D cycle. (**a**) Tomato: chlorosis. (**b**) Eggplant: chlorosis and necrosis. Images recorded on day 21 (tomato) and day 17 (eggplant) of treatment (scale bar = 1 cm).

Eggplants at the two-leaf stage developed leaf chlorosis and necrotic lesions when grown under an 8/4 h L/D cycle in a climate chamber with LED lighting (Figure 1a,c,e and Figure 4b). In contrast, eggplant leaves grown under FLU lamps showed chlorosis but with significantly fewer necrotic spots (Figure 1b,d,f).

Under the same 8/4 h L/D cycle, tomato plants—regardless of the lighting type (LED or FLU)—exhibited interveinal chlorosis (Figure 2 and Figure 4a). Importantly, leaves of both eggplant and tomato plants grown under the standard 16/8 h photoperiod appeared healthy, with no signs of stress-related symptoms.

### 2.3. Photosynthetic Pigments and Chlorophyll Fluorescence

**Figure 5:**
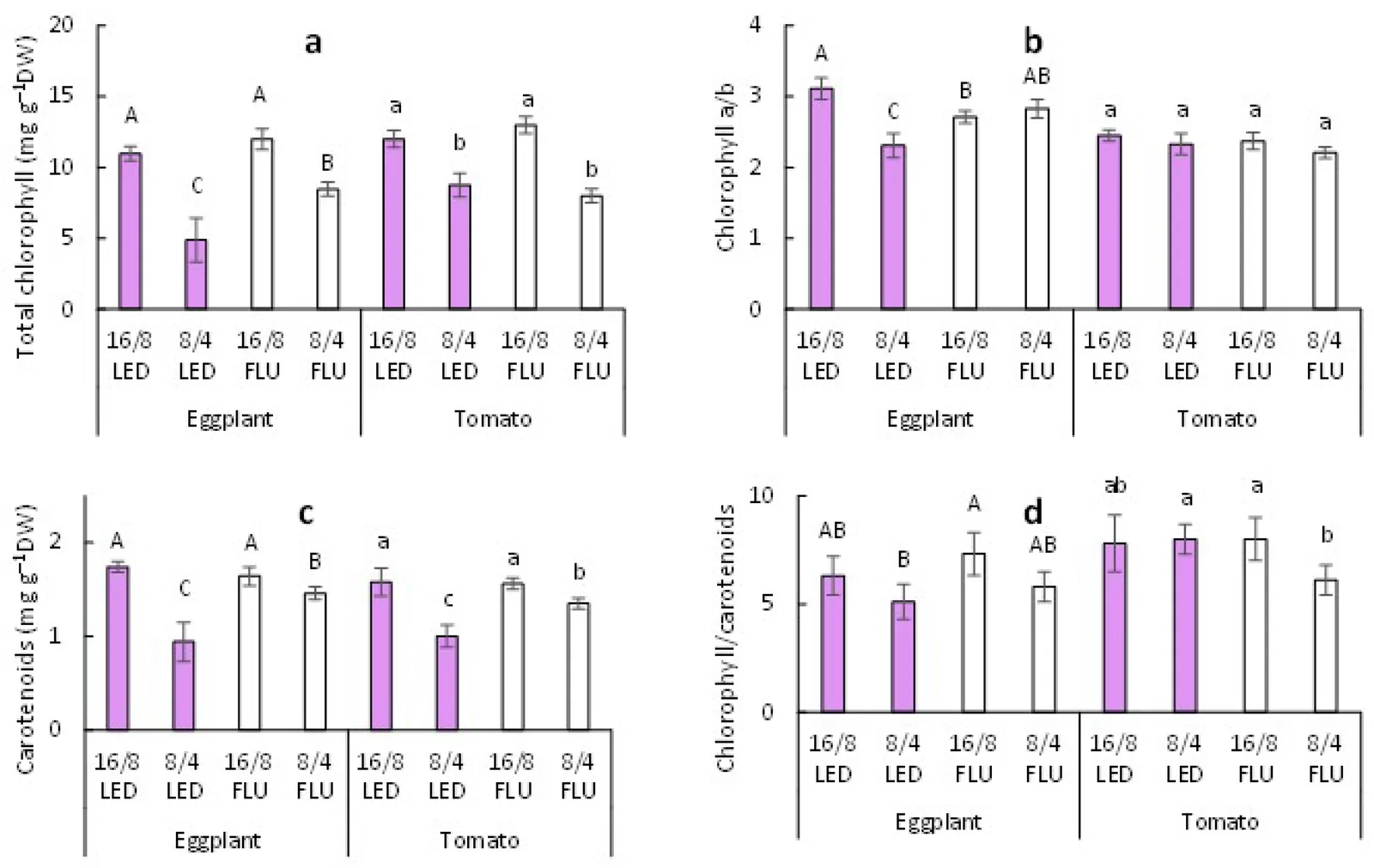
Influence of L/D cycles (16/8 h (control) vs. 8/4 h) and light source type (LED (purple bars) vs. FLU(white bars)) on the content and ratios of photosynthetic pigments in leaves of eggplant (*Solanum melongena* L.) and tomato (*Solanum lycopersicum* L.): (**a**) total chlorophyll content, (**b**) chlorophyll *a*/*b* ratio, (**c**) carotenoid content, (**d**) chlorophyll-to-carotenoid ratio. Statistically significant differences between treatments within each species are denoted by different letters (capital letters for eggplant, small letters for tomato) (*p* < 0.05). Values are expressed as mean ± standard error, *n* = 10.

The 8/4 h L/D cycle significantly reduced chlorophyll *a* + *b* content in both eggplant and tomato leaves when grown under LED or FLU lighting, compared to the standard 16/8 h photoperiod. By the end of the experiment, chlorophyll *a* + *b* content in eggplant leaves had declined by 55% under LED lighting, and by 27–38% across other 8/4 h treatment groups involving both eggplant and tomato plants (Figure 5a).

The chlorophyll *a*/*b* ratio significantly decreased only in eggplants exposed to the 8/4 h L/D cycle under LED lighting (Figure 5b). This change resulted from a more pronounced reduction in chlorophyll *b* content (58%) compared to chlorophyll *a* (48%).

Carotenoid content in both species decreased significantly under the 8/4 h L/D cycle compared to the standard 16/8 h photoperiod (Figure 5c). The greatest reduction was observed in eggplant leaves under LED lighting (46%), followed by tomato leaves under LEDs (37%). Under FLU lamps, the decrease was less marked, with reductions of 11% in eggplant and 13% in tomato.

Exposure to the 8/4 h L/D cycle resulted in a decrease or tendency toward a decrease in the chlorophyll/carotenoid ratio in most cases (Figure 5d). This pattern reflects a more substantial decline in chlorophyll content compared to carotenoids.

**Figure 6:**
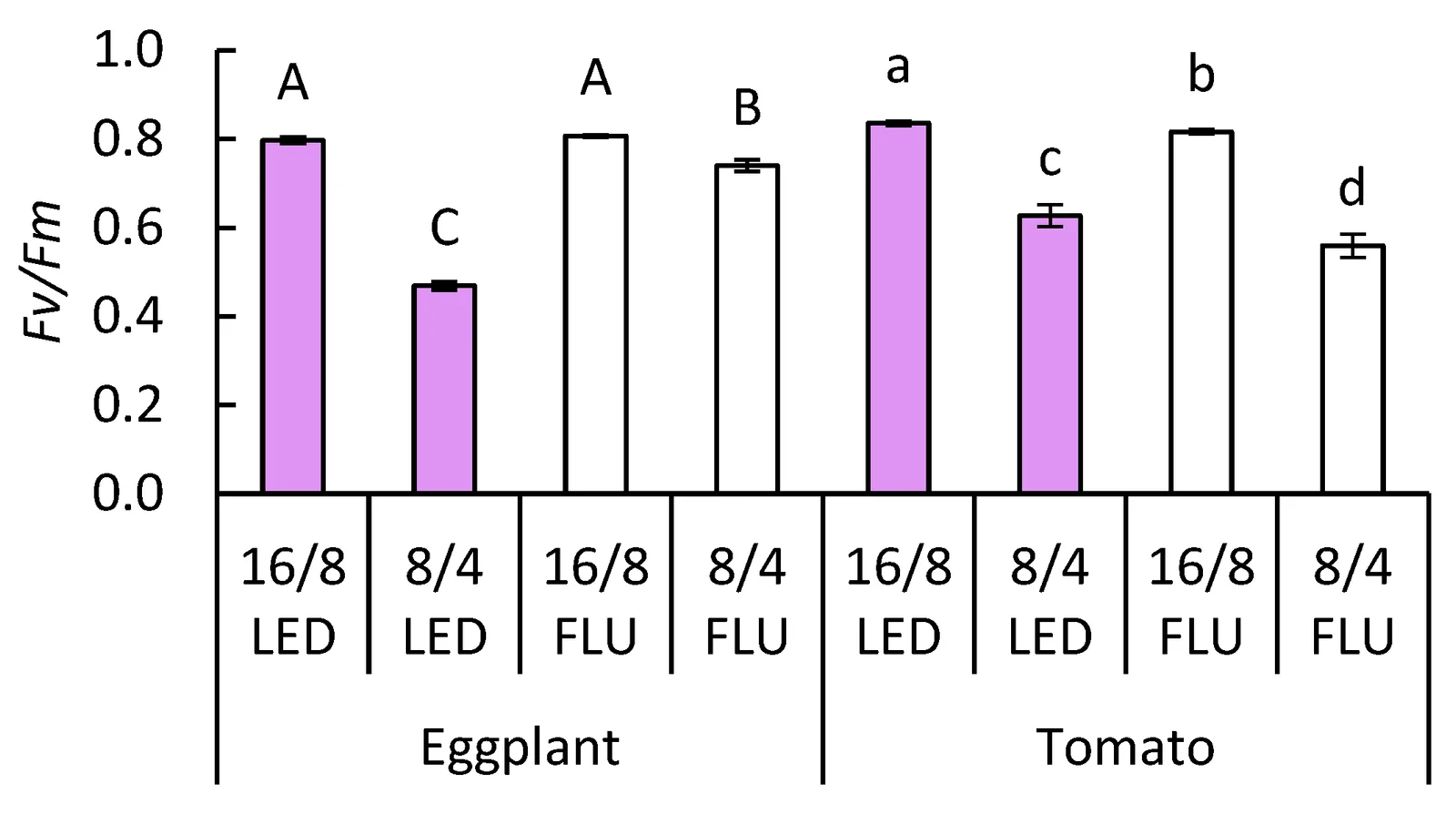
Influence of L/D cycles (16/8 h (control) vs. 8/4 h) and light source type (LED (purple bars) vs. FLU (white bars)) on the maximum quantum yield of photosystem II (*Fv*/*Fm*), a measure of photochemical efficiency, in leaves of eggplant (*Solanum melongena* L.) and tomato (*Solanum lycopersicum* L.). Statistically significant differences between treatments within each species are denoted by different letters (capital letters for eggplant, small letters for tomato) (*p* < 0.05). Values are expressed as mean ± standard error, *n* = 20.

The maximum quantum yield of photosystem II, measured as the *Fv*/*Fm* ratio, consistently exceeded 0.79 in eggplant and tomato plants grown under the standard 16/8 h photoperiod, indicating optimal photosynthetic performance. In contrast, exposure to an 8/4 h L/D cycle led to a significant decline in *Fv*/*Fm* values. Eggplant plants showed *Fv*/*Fm* values of 0.75 under FLU lighting and 0.48 under LED lighting (Figure 6). Tomato plants exhibited values of 0.73 under FLU lighting and 0.57 under LED lighting. These reductions clearly indicate photoinhibition and damage to the photosynthetic apparatus [15], with the most severe effects observed under LED lighting in combination with the 8/4 h L/D cycle.

### 2.4. Oxidative Stress and Antioxidants

**Figure 7:**
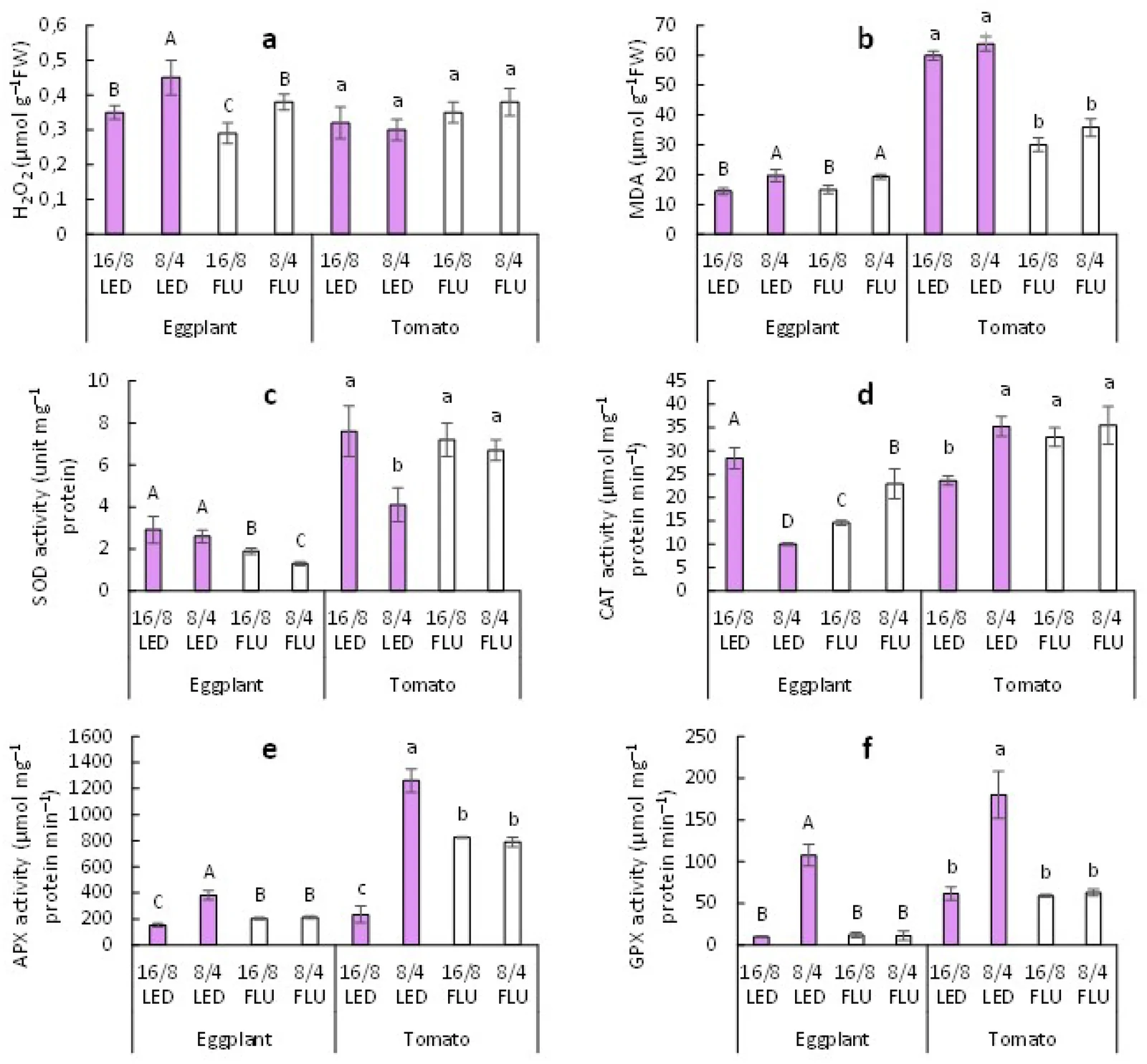
Influence of L/D cycles (16/8 h (control) vs. 8/4 h) and light source type (LED (purple bars) vs. FLU (white bars)) on oxidative stress markers and antioxidative defense system components in leaves of eggplant (*Solanum melongena* L.) and tomato (*Solanum lycopersicum* L.): (**a**) hydrogen peroxide (H_2_O_2_) content, (**b**) malondialdehyde (MDA) content, (**c**) superoxide dismutase (SOD) activity, (**d**) catalase (CAT) activity, (**e**) ascorbate peroxidase (APX) activity, and (**f**) guaiacol peroxidase (GPX) activity. Statistically significant differences between treatments within each species are denoted by different letters (capital letters for eggplant, small letters for tomato) (*p* < 0.05). Values are expressed as mean ± standard error, *n* = 10.

Eggplants exposed to the 8/4 h L/D cycle showed elevated leaf hydrogen peroxide (H_2_O_2_) levels, approximately 30% higher than those in plants grown under the standard 16/8 h photoperiod. This increase occurred regardless of the lighting type (LED or FLU) (Figure 7a). In contrast, tomato plants exhibited no significant differences in H_2_O_2_ content across different lighting regimes or spectra.

A similar pattern emerged for lipid peroxidation, as assessed by malondialdehyde (MDA) content (Figure 7b). In eggplants, MDA levels increased by 37% under 8/4 h LED lighting and by 29% under 8/4 h FLU lighting, compared to the 16/8 h photoperiod (control). In tomato plants, MDA content rose by 7% under LED and by 19% under FLU illumination; however, these changes did not reach statistical significance. These findings suggest that eggplant seedlings are more susceptible to oxidative stress caused by the shortened 8/4 h L/D cycle than tomato seedlings.

Antioxidant enzyme activity was significantly affected by both the shortened L/D cycle and the type of light source. This study did not monitor the dynamics of enzyme activity changes over time; instead, data are presented for a single time point: day 17 for eggplant and day 21 for tomato. Superoxide dismutase (SOD) activity decreased in all plants exposed to the 8/4 h L/D cycle compared to the standard 16/8 h photoperiod (control). However, the reduction was statistically significant only in eggplants grown under 8/4 h FLU lighting and in tomato plants grown under 8/4 h LED lighting (Figure 7c).

Catalase (CAT) activity exhibited species- and treatment-specific responses. In eggplants, CAT activity was significantly reduced by 65% in severely damaged plants grown under the 8/4 h LED regime, compared to those under the standard 16/8 h LED control (Figure 7d). Conversely, under FLU lighting, CAT activity increased by 57% in eggplants exposed to the 8/4 h FLU regime compared to their 16/8 h counterparts.

Ascorbate peroxidase (APX) and guaiacol peroxidase (GPX) activities showed dramatic increases under the 8/4 h LED regimen compared to the standard 16/8 h photoperiod. Specifically: in eggplant leaves, APX activity increased 2.5-fold and GPX activity increased 5.3-fold; in tomato leaves, APX activity increased 10.7-fold and GPX activity increased 2.9-fold. Notably, under FLU illumination, neither APX nor GPX activities changed significantly in response to the shortened L/D cycle in either species.

## 3. Discussion

The study revealed that the abnormal 8/4 h L/D cycle significantly affected the phenotype of eggplant and tomato seedlings. Exposure to this shortened cycle triggered pronounced stress responses, leading to marked reductions in key morphological parameters: plant height, stem diameter, leaf area, and total plant biomass (both FW and DW). Visible symptoms included leaf chlorosis and necrotic lesions. Physiological assessments further confirmed stress induction: a significant reduction in the *Fv*/*Fm*, indicating photoinhibition; decreased total chlorophyll content; elevated levels of oxidative stress markers (hydrogen peroxide and MDA, a product of lipid peroxidation); increased activity of antioxidant enzymes, particularly GPX and APX, reflecting the activation of defense mechanisms. These findings demonstrate that the shortened L/D cycle disrupts normal growth and induces oxidative stress in both species, with eggplants exhibiting more severe symptoms than tomatoes. This aligns with previous results obtained for two-week-old tomato plants exposed to other shortened L/D cycles (9/3 h, 6/3 h, and 2/1 h) [12]. These studies revealed similar stress responses across treatments. However, fewer data points are available for eggplant, which limits direct comparison with other species. Specifically, it is known that exposing two-week-old eggplant seedlings to a short 6/6 h L/D cycle induces leaf chlorosis and causes a significant decrease in growth performance [11].

The stress response observed in plants exposed to the 8/4 h L/D cycle cannot be explained by differences in traditional light parameters. Light intensity was identical for all plants within each treatment group. Daily light integral (DLI) was equal in both treatments (16/8 h and 8/4 h), since plants received 16 h of illumination per day. Spectral composition remained constant across all experimental series, with both LED and FLU light sources used consistently. Therefore, the three key light parameters typically considered in plant light response studies—light intensity, photoperiod (total daily light duration), and spectral composition—were kept constant. Under these controlled conditions, plants under the standard 16/8 h photoperiod remained healthy, whereas those exposed to the 8/4 h cycle showed visible signs of stress and damage. The applied experimental treatment modulated both the overall cycle duration and the frequency of L/D transitions per day, concurrently altering the uninterrupted light and dark phases. However, the current experimental design precludes the isolation of these individual effects. The data suggest that the observed stress response is attributable to the temporal redistribution of light and darkness, as opposed to the total light dose or spectral composition.

Abnormal L/D cycles—both shortened and extended—have recently gained increasing use not only in scientific experiments but also in practical applications. Over the past two decades, compelling evidence has accumulated showing that such cycles can significantly impact plant growth and development. Importantly, their effects may be either negative or positive, depending on the specific L/D cycle parameters and plant sensitivity [4,5,6,7]. For instance, using different L/D regimes—such as a standard 16/8 h photoperiod, two 8/4 h cycles, four 4/2 h cycles, or even eight 2/1 h cycles—can yield plants with distinct phenotypes and varying levels of nutrients or functional substances. In certain cases, this enhances the consumer appeal and/or market value of plant products [8,9,10,11]. Other parameters of the light regime—the DLI (light intensity × total duration of the light phase) and the spectral composition—remain unchanged. The collective evidence suggests that conventional three-parameter models of light exposure fail to adequately describe plant responses under non-standard L/D cycles. Critical supplementary factors—notably cycle duration, frequency of L/D transitions, and the lengths of continuous (uninterrupted) light and dark phases—exert substantial influence on plant growth and developmental processes. These factors do not manifest under natural conditions. This is because a typical 24 h diurnal cycle consistently features a single, predictable transition from day to night and back, with no intermediate transitions or variations in cycle period.

Recent findings indicate that subjecting plants to abnormal L/D cycles under artificial illumination—as opposed to the natural 24 h photoperiod—generates a desynchronization between the plant’s endogenous circadian clock and the imposed light environment. This desynchronization constitutes the primary cause of the physiological stress response commonly referred to as photoperiod stress [16,17,18,19]. Photoperiod stress (termed circadian stress by the authors) was first identified in cytokinin-deficient *Arabidopsis* plants and in the circadian clock mutants lacking clock genes such as CCA1 (CIRCADIAN CLOCK ASSOCIATED1), LHY (LATE ELONGATED HYPOCOTYL), and ELF3 (EARLY FLOWERING3) [16,17,20,21]. Mutant plants showed a strong molecular response, which then manifested phenotypically as necrotic lesions on the leaves of the plants, which ultimately died. It was later shown that the regulators of the stress response in plants are cytokinins (mainly trans-zeatin, which performs a protective function acting through the receptor ARABIDOPSIS HISTIDINE KINASE3 (AHK3) and the transcriptional regulators ARABIDOPSIS RESPONSE REGULATOR2 (ARR2), ARR10 and ARR12) and the CCA1/LHY proteins, which are two key regulators of the biological clock responsible for maintaining circadian rhythms under continuous lighting. This indicates that the normal functioning of the circadian clock is necessary for the plant to overcome photoperiodic stress, and cytokinin helps to maintain the function of the internal timer, especially under stress [16,20].

Should the stress in our study be attributed to the redistribution of a 16 h daily illumination into two 8/4 h L/D cycles, this would suggest that eggplant and tomato plants were exposed to photoperiodic stress. Given that circadian clock gene expression, rhythmic physiological processes, hormonal dynamics, and phase markers were not measured in the present experiment, we advance the hypothesis that the observed injuries may result from circadian desynchronization. This indicates that photoperiodic stress is not limited to mutant plants but can also occur in normal plants sensitive to circadian asynchrony. Eggplant and tomato serve as clear examples of such sensitive species [22,23]. We propose that, similar to other types of stress, plant species exhibit varying degrees of sensitivity or tolerance to circadian asynchrony. Notably, this pattern is already observed in responses to continuous lighting—an extreme form of abnormal L/D cycle—where both species-specific and varietal-specific differences have been documented [24,25,26,27].

The parameters of shortened and extended L/D cycles—including cycle length and light-to-dark ratio—as well as associated conditions (such as light intensity and spectral composition), are critical determinants of plant responses. Studies report both positive and negative effects under such conditions. In lettuce, comparative analyses between standard photoperiods (16/8 h and 8/16 h) and shortened cycles with equivalent DLI—such as 9/3 h, 6/2 h, 4/8 h, and 2.67/5.3 h—demonstrated that a single L/D cycle per day yields better results than multiple cycles. Specifically, lettuce yield and quality were significantly higher under standard photoperiods [28,29]. Using short L/D cycles in these experiments altered transpiration rates and caused fluctuations in carbohydrate and metabolite levels, disrupting the normal daily synchronization of physiological processes. The studies demonstrate that no single “ideal” photoperiodic regime exists. Instead, different combinations of L/D cycles and light intensities can optimize specific objectives, such as shoot biomass maximization; root development; secondary metabolite accumulation (for example, anthocyanins); photosynthesis efficiency. Notably, replacing a single 12/12 h L/D cycle with multiple shorter cycles (6/6 h, 4/4 h, or 3/3 h) significantly impaired biomass accumulation, leaf expansion, and photosynthetic performance in lettuce [30,31]. In contrast, lettuce yield was significantly higher under shortened L/D cycles (4/2 h, 3/1.5 h, and 2/1 h) compared with the standard 16/8 h photoperiod [5]. This study employed intermittent exposure to alternating red and blue light, which proved to be an effective technique. It not only stimulated plant growth but also altered the carbohydrate profile, leading to increased sweetness in the lettuce. Furthermore, a 6/6 h photoperiod significantly reduced dry weight, plant height, and leaf area in tomato and cucumber plants compared with a 12/12 h photoperiod [32]. However, hot pepper plants showed unchanged biomass under a 6/6 h L/D cycle. The author notes that, although shortened L/D cycles may reduce overall nutrient uptake, this approach remains viable for specific applications, particularly in indoor farming setups where minimizing lighting energy costs is a priority. Balancing light levels is critical; excessive reduction can lead to nutrient deficiencies, while moderate reductions, combined with proper nutrient management, offer a practical compromise between energy savings and productivity. Another study revealed that 9/3 h and 6/2 h photoperiods had no significant effect on tomato seedling growth. In contrast, cucumber yield was significantly reduced compared with an 18/6 h photoperiod [33]. The authors partially attributed the differing responses of tomato and cucumber plants to variations in phytochrome A concentration—a key regulator of photoperiodic responses. Short L/D cycles had contrasting effects across species: in celery (4/2 h) [34] and stevia (5.3/2.7 h) [35], growth was significantly suppressed. In grapes, however, a 3/3 h cycle enhanced photosynthetic efficiency and promoted growth compared with the standard 12/12 h photoperiod [36]. Additionally, basil plants demonstrated tolerance to intermittent lighting regimes [4]. These findings highlight that responses to abnormal L/D cycles are highly species-specific and may vary considerably even among different cultivars of the same species.

In this study, stress responses to shortened L/D cycles were more pronounced in both species under LED lighting compared with FLU conditions. These results corroborate earlier findings, integrating intermittent illumination with adjustments in light intensity or quality elicits distinct physiological responses, underscoring the complex interplay between lighting regimes and plant performance [9]. Comparative analysis in lettuce [37] showed that LED lighting induces more pronounced changes in plant responses to photoperiod variations than FLU lamps, with the strongest effect observed under red LED illumination. Furthermore, LEDs allow more precise control of key parameters such as light intensity and wavelength than FLU lamps. As a result, they enable the delivery of pulsed or sinusoidal light signals to modulate circadian rhythms [38]. By precisely regulating spectral composition, light intensity, L/D cycle length, and DLI distribution across the 24 h cycle, we can modulate the light environment to alleviate plant stress responses. This approach better synchronizes artificial lighting with endogenous biological rhythms, promoting optimal growth conditions [9,39]. In our experiments, LED and FLU lamps were housed in different chambers. Therefore, the stronger response to abnormal L/D cycle under LED illumination cannot be attributed solely to the light source.

## 4. Materials and Methods

### 4.1. Plant Material and Growth Conditions

Seeds of eggplant (*Solanum melongena* L. cv. Almaz) and tomato (*Solanum lycopersicum* L., cv. Verlioka Plus) were surface-sterilized, germinated, and transplanted into individual plastic containers filled with a commercial soil mixture. All seeds were procured from OOO Gavrish (Moscow, Russia). Plants were grown under controlled environmental conditions in two types of growth chambers: (1) Vötsch chamber (Vötsch Industrietechnik, Balingen, Germany) equipped with fluorescent (FLU) lamps: OSRAM L36W/77 FLUORA (7700 K, cool daylight); OSRAM L36W/830 LUMILUX Warm White (3000 K, warm white); OSRAM L36W/765 LUMILUX Cool White (6500 K, neutral white) (OSRAM GmbH, Munich, Germany); (2) BJPX-RG-P160C chamber (Biobase, Jinan, China) equipped with broad-spectrum LED modules (6550 K, spectral range 400–800 nm).

Environmental conditions were maintained as follows: air temperature 23 °C, relative humidity 70%, photosynthetic photon flux density (PPFD) 200 ± 25 μmol m^−2^ s^−1^, measured at plant canopy level using a LI-250A Light Meter (Li-COR Biosciences, Lincoln, NE, USA). Plants were irrigated daily to maintain optimal soil moisture.

The experimental design included two distinct L/D cycle regimes: (1) Standard regime (16 h of light followed by 8 h of darkness (16/8 h), representing a conventional photoperiod); (2) Shortened L/D cycle regime (8 h of light followed by 4 h of darkness (8/4 h), repeated twice within a 24 h period) with two light sources (LED and FLU). DLI was maintained at 11.5 mol m^−2^ day^−1^ for both treatments. This ensured that the total amount of light received per day was equivalent between the regimes, differing only in temporal distribution. Plants were rotated daily within the chambers to ensure uniform light exposure.

Eggplant seedlings were exposed to the respective L/D cycles for a period of 17 days, while tomato seedlings underwent the treatment for 21 days to account for species-specific growth rates. All measurements and sampling were performed at a consistent time point: 1 h after lights were turned on for each treatment. Measurements were taken on the second true leaf.

### 4.2. Growth Measurements

Plant height, shoot diameter at the cotyledon node, leaf area, and fresh weight (FW) of the aboveground parts were measured (*n* = 10). To minimize evaporation losses, plants were excised at the soil level and weighed immediately for FW determination. Leaves were then separated from stems and dried separately at 105 °C until a constant dry weight (DW) was achieved. Prior to drying, leaves were scanned, and their area was quantified using the AreaS 2.1 software (developed by Permyakov A.N.). Leaf mass per area (LMA) was calculated as the ratio of the dry mass of lamina disks to their area. For this, eight 8 mm diameter disks were punched from each leaf using a cork borer.

### 4.3. Photosynthetic Pigment Content

Pigment extraction and quantification were performed as follows: chlorophyll *a*, chlorophyll *b*, and total carotenoids were extracted in 96% ethanol. Absorbance was measured at specific wavelengths (665 nm, 649 nm, and 470 nm) using an SF2000 spectrophotometer (Spectrum, St. Petersburg, Russia) (*n* = 5). Pigment concentrations were then calculated using the established spectrophotometric equations for ethanol extracts [40]. Concentrations are expressed in mg g^−1^ DW.

### 4.4. Chlorophyll Fluorescence Measurements

Chlorophyll fluorescence parameters were measured using a Pulse Amplitude Modulation fluorometer MINI-PAM II (Heinz Walz GmbH, Effeltrich, Germany). The potential quantum yield of photochemical activity of photosystem II (*Fv*/*Fm*) was determined after a 20 min dark adaptation period. Leaves were dark-adapted using leaf clips (*n* = 10).

### 4.5. Malondialdehyde (MDA) Content

The content of malondialdehyde (MDA), a marker of lipid peroxidation, was determined using the thiobarbituric acid reactive substances (TBARS) assay. This standard method is based on the reaction of MDA with thiobarbituric acid, forming a trimethine complex with an absorption maximum at 532 nm [41]. To account for nonspecific absorption, absorbance at 600 nm was measured for each sample and subtracted from the absorbance value at 532 nm (*n* = 5). MDA concentration was calculated using an extinction coefficient of 155 mM^−1^cm^−1^. Lipid peroxidation levels were expressed as micromoles of MDA per gram of FW (μmol g^−1^ FW).

### 4.6. Hydrogen Peroxide Content

Hydrogen peroxide (H_2_O_2_) content was quantified according to Velikova et al. [42]. Briefly, 0.1 g leaf tissue was homogenized in 2 mL 0.1% trichloroacetic acid on ice, centrifuged (12 000× *g*, 15 min, 4 °C), and 0.5 mL supernatant was mixed with 0.5 mL phosphate buffer (pH 7.0) and 1 mL 1 M KI. Absorbance was measured at 390 nm, and H_2_O_2_ concentration was determined from a calibration curve (μmol g^−1^ FW) (*n* = 5).

### 4.7. Antioxidative Enzyme Activity Assays

The activities of key antioxidant enzymes were determined in leaf extracts using an SF-2000 spectrophotometer (*n* = 5). Catalase (CAT, EC 1.11.1.6) activity was measured by monitoring the rate of hydrogen peroxide (H_2_O_2_) decomposition at 240 nm. The decrease in absorbance was recorded over a 1 min period, and CAT activity was expressed as μmol H_2_O_2_ min^−1^ mg^−1^ protein [43]. Superoxide dismutase (SOD, EC 1.15.1.1) activity was determined by its ability to inhibit the photochemical reduction of nitro blue tetrazolium (NBT) to formazan. One unit of SOD activity was defined as the amount of enzyme causing 50% inhibition of NBT reduction under illumination [44]. Ascorbate peroxidase (APX, EC 1.11.1.11) activity was assayed by monitoring the oxidation of ascorbic acid at 290 nm in the presence of exogenous H_2_O_2_. The reaction mixture contained ascorbic acid, H_2_O_2_, and enzyme extract in phosphate buffer (pH 7.0) [45]. Guaiacol peroxidase (GPX, EC 1.11.1.7): activity was quantified by measuring the increase in absorbance at 470 nm due to the oxidation of guaiacol in the presence of H_2_O_2_. The extinction coefficient of tetraguaiacol (ε = 26.6 mM^−1^ cm^−1^) was used for calculation [46].

Enzyme activities were normalized to protein content, which was determined according to the Bradford method [47] using bovine serum albumin (BSA) as a standard.

### 4.8. Data Analysis

The experiment was conducted in two independent runs using a completely randomized design, with 20 seedlings per treatment per run. For measurements, 10 samples were collected for non-destructive analyses and 5 samples for destructive analyses. To assess the effects of L/D cycles (16/8 h and 8/4 h), light source (LED and FLU), and their interaction, data from both runs were pooled and analyzed using two-way ANOVA, followed by post hoc comparisons with the Tukey HSD test (significance level *p* < 0.05). Data are presented as mean ± standard error.

## 5. Conclusions

Redistributing the same 16 h daily illumination into two 8/4 h L/D cycles induced substantial morphological, photosystem II (PSII), and redox responses under the tested conditions. The shortened 8/4 h L/D cycle resulted in significant growth suppression, reduced leaf area, and decreased maximum quantum yield of photosystem II (*Fv*/*Fm*), as well as chlorophyll and carotenoid content in both species. Under this regime, eggplants exhibited leaf chlorosis accompanied by necrotic lesions, whereas tomato plants displayed interveinal chlorosis. Notably, a significant accumulation of oxidative stress markers—a 37% increase in MDA and a 30% elevation in hydrogen peroxide (H_2_O_2_) levels—occurred exclusively in eggplant. These findings demonstrate that the shortened L/D regimen induces stress in both species, with eggplant exhibiting greater injury than tomato.

The data obtained, combined with literature analysis, suggest that conventional three-parameter models of light exposure fail to adequately describe plant responses under abnormal L/D cycles. Critical supplementary factors—notably cycle duration, frequency of L/D transitions, and the lengths of continuous (uninterrupted) light and dark phases—exert substantial influence on plant growth and developmental processes. However, the current experimental design precludes the isolation of these individual effects. The results indicate that the observed stress response is attributable primarily to the temporal redistribution of light and darkness, rather than to the total light dose or spectral composition.

Further research is required to elucidate the underlying circadian mechanisms involved in the differential stress response between eggplant and tomato; validate the apparent differences in plant performance under LED versus FLU lighting conditions through controlled experiments; develop more comprehensive models of light exposure that incorporate temporal parameters for optimizing crop production in controlled-environment agriculture systems, such as vertical farms.

## Figures and Tables

**Table 1:** Results of two-way ANOVA for all dependent variables measured in eggplant (*Solanum melongena* L.) cv. ‘Almaz’.

Parameter	Treatment Factor, Interaction		
	Light Source	L/D Cycle	Light Source × L/D Cycle
Plant height	ns	***	ns
Stem diameter	***	*	ns
Leaf area	***	ns	ns
LMA	ns	***	*
Shoot fresh weight	***	ns	ns
Shoot dry weight	***	ns	ns
Total chlorophyll	*	***	*
Carotenoids	*	***	*
*Fv*/*Fm*	*	*	***
H_2_O_2_	*	***	*
MDA	*	***	ns
SOD activity	***	ns	*
CAT activity	***	*	ns
APX activity	*	ns	ns
GPX activity	*	ns	ns

Here and in Table 2: Asterisks denote significance levels: * *p* < 0.05, *** *p* < 0.001; ns, not significant.

**Table 2:** Results of two-way ANOVA for all dependent variables measured in tomato (*Solanum lycopersicum* L.) cv. ‘Verlioka Plus’.

Parameter	Treatment Factor, Interaction		
	Light Source	L/D Cycle	Light Source × L/D Cycle
Plant height	***	***	*
Stem diameter	ns	ns	***
Leaf area	ns	*	***
LMA	***	***	***
Shoot fresh weight	*	***	*
Shoot dry weight	***	***	***
Total chlorophyll	*	*	ns
Carotenoids	*	***	*
*Fv*/*Fm*	*	*	***
H_2_O_2_	ns	*	ns
MDA	***	ns	ns
SOD activity	ns	ns	*
CAT activity	ns	ns	ns
APX activity	ns	ns	ns
GPX activity	ns	ns	ns

## Data Availability

The data presented in this study are available on request from the corresponding author.
